# Dual-Stack Single-Radio Communication Architecture for UAV Acting As a Mobile Node to Collect Data in WSNs

**DOI:** 10.3390/s150923376

**Published:** 2015-09-16

**Authors:** Ali Sayyed, Gustavo Medeiros de Araújo, João Paulo Bodanese, Leandro Buss Becker

**Affiliations:** 1 Department of Automation and Systems, Federal University of Santa Catarina, Florianópolis 88040-900, Brazil; E-Mails: ali.sayyed@hotmail.com (A.S.); joao.bodanese@gmail.com (J.P.B.); 2 Campus Araranguá, Federal University of Santa Catarina, Araranguá 88906-072, Brazil; E-Mail: gustavo.araujo@ufsc.br; 3 Instituto Senai de Sistemas Embarcados, Florianópolis 88040-900, Brazil

**Keywords:** mobile nodes, unmanned aerial vehicles, communication architecture, data collection, wireless sensor networks

## Abstract

The use of mobile nodes to collect data in a Wireless Sensor Network (WSN) has gained special attention over the last years. Some researchers explore the use of Unmanned Aerial Vehicles (UAVs) as mobile node for such data-collection purposes. Analyzing these works, it is apparent that mobile nodes used in such scenarios are typically equipped with at least two different radio interfaces. The present work presents a Dual-Stack Single-Radio Communication Architecture (DSSRCA), which allows a UAV to communicate in a bidirectional manner with a WSN and a Sink node. The proposed architecture was specifically designed to support different network QoS requirements, such as best-effort and more reliable communications, attending both UAV-to-WSN and UAV-to-Sink communications needs. DSSRCA was implemented and tested on a real UAV, as detailed in this paper. This paper also includes a simulation analysis that addresses bandwidth consumption in an environmental monitoring application scenario. It includes an analysis of the data gathering rate that can be achieved considering different UAV flight speeds. Obtained results show the viability of using a single radio transmitter for collecting data from the WSN and forwarding such data to the Sink node.

## 1. Introduction

Over the last 15 years, Wireless Sensor Networks (WSNs) have been studied extensively and applied in a large number of real world applications, ranging from environment monitoring (e.g., pollution, agriculture, volcanoes, structures and buildings health), to event detection (e.g., intrusions, fire and flood emergencies) and target tracking (e.g., surveillance). WSNs usually generate a large amount of data by sensing their environment and detecting events, that must be managed and forwarded to a Sink node in an efficient and reliable manner.

The introduction of mobility in WSN has attracted significant interest in recent years [1]. Mobility can be introduced in any component of a sensor network, including the regular sensor nodes, relay nodes (if any), data collectors, sink or any combination of these. From nodes deployment and localization to data routing and dissemination, mobility plays a key role in almost every operation of sensor networks. For instance, a mobile node can visit other nodes in the network and collect data directly through single-hop transmissions [2]. Similarly, a mobile node can move around the sensor network and collect messages from sensors, buffer them, and then transfer them to the Sink [3]. This significantly reduces not only message collisions and losses, but also minimizes the burden of data forwarding by nodes, and as a result spreads the energy consumption more uniformly throughout the network [1]. Mobility can also improve WSNs in terms of extending lifetime [4], coverage [5], reliability [6], and channel capacity [7]. A detailed survey on the usage and advantages of mobility in different phases of the WSN operation is given in [8].

Unmanned Aerial Vehicles (UAVs) are increasingly being used in WSNs, due to the cost-effective wireless communication and surveillance capabilities they provide [9]. UAVs play a prominent role in a variety of real world applications like homeland defense and security, natural disaster recovery, real-time surveillance, among others. UAVs and WSNs can cooperate in a number of different ways to improve network efficiency and performance [10,11,12,13,14]. Benefits can be identified in both directions. For instance, UAVs that have more resources for sensing and computing, can move to particular locations to perform more complicated missions. In addition, the WSN could provide extended sensory capabilities to guide the navigation of UAVs.

In order to support simultaneous interaction with WSN and Sink, the UAV must have at least two types of communication protocols. As sensor nodes have constrained resources, their communications protocol should be efficient and have a small footprint. This holds for communication among the WSN nodes as well as WSN-to-UAV and UAV-to-WSN. When considering communication between the UAV and the Sink, more robust protocols can be adopted, given that both sides have much more computing power when compared to the nodes in a WSN. In order to support UAV-to-Sink communication, there are two possibilities: either the UAV carries a second radio (with different physical layer) or it uses the same radio for both purposes. Most related works analyzed so far make use of two or even three radios for such purposes, as further discussed.

In this paper, we detail the Dual-Stack Single-Radio Communication Architecture (DSSRCA), which was first presented in [15]. DSSRCA allows a mobile node (e.g., a UAV) to communicate in a bidirectional manner with a WSN and a Sink node using the same radio. This architecture is composed of a dual stack protocol designed specifically to meet the different network QoS requirements present in UAV-to-WSN and UAV-to-Sink communications, such as best-effort and more reliable communication. DSSRCA suits situations where the mobile node acts as data collector for the WSN.

Obtained results confirm the viability of using a single radio transmitter for collecting data from the WSN and flushing such data back to a Sink node, or even for sending telemetry data to a Base Station. The designed prototype allowed a real UAV to collect data from heterogeneous ground sensor devices using best-effort communication and send data back to the Sink using reliable communication. This paper also includes a simulation analysis that observes the bandwidth consumption in an environmental monitoring application scenario.

The remaining parts of this paper are organized as follows. Section 2 presents and discusses some related works, including different schemes proposed for using mobile data collectors in WSNs. In Section 3, the proposed Dual-Stack Single-Radio Communication Architecture (DSSRCA) is explained, along with the hardware and software components, and some experimental results with a real UAV. Next, in Section 4, a detailed simulation study related to bandwidth consumption in an environmental monitoring application scenario is presented and discussed. Finally, conclusions and future work directions are addressed in Section 5.

## 2. Related Works

A considerable number of approaches exploiting mobility for data collection in WSNs have been proposed in recent years. Mobility can be introduced in any component of the WSN, including regular sensor nodes, relay nodes (if any), data collectors, sink or any combination of these. For instance, in the case of *mobile sensor nodes*, as shown in [16,17], animals / people with attached sensors, moving in the network field, not only generate their own data, but also carry and forward data coming from other nodes that they have been previously in contact with. These mobile sensors eventually transfer all their data when in contact with the sink node. Similarly in case of *mobile sink*, as shown in [4,18,19], the overall energy consumption of the network is minimized by changing the position of the sink node which collects data from sensor nodes either directly (*i.e.*, by visiting each of them) or indirectly (*i.e.*, through relays or other nodes). Finally, *mobile data collectors*, which are neither producers nor consumers of messages in a sensor network, perform the specific tasks of collecting data from sensor nodes when in their coverage range and eventually passing it in to the Sink. In this paper we focus mainly on approaches based on mobile data collectors (MDCs), because in our application scenario the UAV acts as a MDC that moves in the network area with predictable / deterministic mobility patterns while collecting data from the underlying ground nodes.

### 2.1. Data Collection Schemes Based on Mobile Data Collector

In this case, data is buffered at the source nodes until a mobile data collector visits and collects data over a single-hop wireless transmission. Existing proposals in this category can be further classified in three categories according to the mobility patterns of the mobile data collector [20,21]. *i.e.*, *Random mobility*, *Predictable mobility* and *Controlled mobility*.

In the rest of this sub section, we present a brief overview of the state-of-the-art in this regard. Interested readers should refer to [21] for a detailed survey.

#### 2.1.1. Mobile Data Collectors with Random Mobility

The concept of mobile data collector was first introduced in [3], in which MDCs are referred to as Data Mules. In this proposal, generated data is buffered at source sensors until MDCs move randomly (using a Markov model based on a two-dimensional random walk) and collect data opportunistically from sensors in their direct communication range. The MDCs then carry the collected data and forward it to a set of access points. In this case, static nodes periodically wake up and listen for advertisements from mobile node for a short time. If it does not hear any beacon message from a mobile node, it return to sleep. Otherwise it starts transferring data to the mobile node.

An efficient Data Driven Routing Protocol (DDRP) was proposed in [22] where mobile sinks periodically broadcast beacon messages to their one-hop neighbors as they move around the network area. Each node maintains a variable called Dist2Sink (Distance to Sink), which stores the shortest number of hops to the sink. Beyond a certain value, Dist2Sink is equal to infinity, which means the node has no route to the sink. When a node’s Dist2Sink variable is equal to infinity, it waits for a certain amount of time to overhear about a new valid route to the sink. However, if unsuccessful within a time bound, nodes adopt a random-walk strategy until the packet finds a valid route to the sink or is timed out and dropped. The main problem with DDRP is that with each movement of the mobile sink, subsequent topological changes occur that are propagated in the entire network in the form of the overhearing mechanism.

Safdar *et al.* proposed an improvement in [23] for Routing Protocol for Low Power and Lossy Networks (RPL) to efficiently support sink mobility. In case of basic RPL, mobile sink frequently broadcasts its presence which is propagated in the entire sensor field and each node in the network makes a Directed Acyclic Graph (DAG) to the sink. This promotes extensive network traffic and high energy consumption with each movement of the sink. However, in [23] the sink’s topological update is restricted to a confined zone, consisting of a few hops around the sink. Hence, nodes within the confined zone can immediately send data to the sink using the known DAGs. The size of confined zones is increased for low sink mobility and decreased for high sink mobility. Nodes outside the confined zones implement on demand sink discovery. With each unsuccessful attempt, the zone size for broadcasting the route discovery request is increased. This procedure is repeated until a network wide broadcast for route discovery is initiated.

#### 2.1.2. Mobile Data Collectors with Predictable or Deterministic Mobility

In this case, the static and mobile nodes agree on a specific time at which the data transfer may initiate. Mobile nodes follow a very strict schedule and other nodes know exactly when mobile nodes will be in their communication range. For instance, in [24], mobile nodes are assumed to be on board of public transportation shuttles that visit sensor nodes according to a schedule. In this way the sensor nodes calculate the exact active time and wake up accordingly to transfer data.

In [25] a scheme called Multiple Enhanced Specified-deployed Sub-sinks (MESS) for WSNs is proposed for data collection using multiple sub-sinks. The sub-sinks are considered as enhanced nodes having more resources and deployed at equal distances along the accessible path, creating a strip in the sensing field and providing a set of meeting points with the mobile sink. Each sub-sink working as an access-point to the mobile sink notifies the underlined network segment about the service it is offering.

Similarly in [26] Oliveira *et al.* proposed a greedy algorithm called Wireless HIgh SPEed Routing (Whisper) to forward data to a high speed sink. The scheme is based on the assumption that all nodes know their own locations, their neighbors’ locations, and the path and displacement of the mobile sink. In this case, the sink, moving at high speed, does not stay permanently at the location of interest, and therefore refreshes its future location based on its speed and trajectory. The interested sensor nodes cannot directly send message to the fast moving sink and therefore forward their data towards the estimated meeting point with the sink. The meeting point is estimated on the basis of various delays in message transmission together with the node’s own location, the neighbors’ locations, and the estimated sink location.

#### 2.1.3. Mobile Data Collectors with Controlled Mobility

In some WSNs data collection is performed only when events of interest occurs. In this case, controlled mobility is desired since it allows triggering mobile nodes on demand to visit sensors and avoiding sensor buffer overflows. For instance, in [9] the problem is proved to be NP-complete and a heuristic solution called Earliest Deadline First (EDF) and its two variants are presented. With the EDF solution, the next node to be visited by the mobile node is chosen as the one that has the earliest buffer overflow deadline.

Banerjee *et al.* proposed a scheme in [27], in which multiple resource-rich mobile cluster heads (CHs) are employed to prolong network lifetime and ensure delay requirements of real time applications are met. In this case, mobile cluster heads cooperatively collect data from different network segments and deliver it to a central Sink. All CHs move in a manner ensuring their connectivity with the base station while covering most interested areas at the same time. The authors propose three different movement strategies for CHs in order to reduce multi-hop communication and enhance network lifetime.

Similarly, authors in [7] used different controlled mobility approaches to optimize the speed of mobile nodes while collecting data in a sensor network. The first approach is called stop and communicate because as the mobile node enters the communication range of a static node that has some data to send, it stops there until all buffered data has been collected. The duration of the stop depends on the data generation rate of the source node. The second way to optimize speed is called adaptive speed control in which the speed of mobile node is changed according to the number of encountered nodes and the percentage of collected data with respect to buffered messages. Different groups of nodes are made according to the amount of data collected from them (low, medium or high). The mobile node moves slowly in a group with a low collected data percentage, while it moves faster when it is in the communication range of nodes with a high percentage of collected data.

### 2.2. Preliminary Conclusions

There are numerous other mobility based data collection approaches that target one aspect of data collection or another and have different pros and cons. After analyzing all the state-of-the-art solutions regarding mobile data collectors it is possible to conclude that:

– Approaches using a single radio in the mobile data collector only perform data collection, *i.e.*, the mobile node does not communicate with the sink node while on the quest for data collection.
– Approaches targeting simultaneous communication with sensor nodes and sink or base station use at least two different radios on the mobile data collector.

This motivated us to design an architecture that can communicate with both the WSN and the sink node using the same radio. Our work is different in the way that it focuses on using a dual stack that allows a mobile node, in our case a UAV, with deterministic mobility patterns, to collect data from heterogeneous ground sensor nodes and send telemetry or collected data back to the Sink node using the same communication hardware and without compromising the data collection rate. The details about this architecture are presented in the next section.

## 3. Dual-Stack Single-Radio Communication Architecture (DSSRCA)

**Figure 1:**
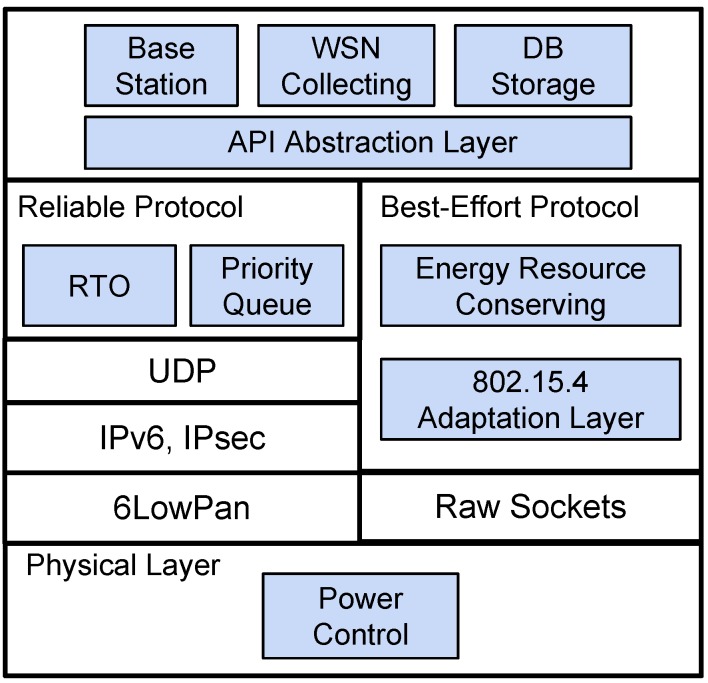
Dual-Stack Single-Radio Communication Architecture (DSSRCA) overview: customized transport layer on top of 6LoWPAN (**left**) and the small-footprint Wireless Sensor Network (WSN) protocol (**right**).

The proposed Dual-Stack Single-Radio Communication Architecture (DSSRCA) aims to provide a flexible communication infrastructure that supports UAV-to-WSN and UAV-to-Sink communications using a single radio. It is able to commute between best-effort and reliable communication strategies according to the different network QoS requirements present in UAV-to-WSN and UAV-to-Sink communications. An overview of DSSRCA organization in terms of partitions, layers, and sub-components is illustrated in Figure 1.

The top layer in Figure 1 refers to the application level, which could be either at the Sink node or at the mobile data collector (MDC). On the left, it is the customized transport layer on top of 6LoWPAN that constitutes the reliable protocol used in UAV-to-Sink communication. Finally, the small-footprint best-effort protocol used for UAV-to-WSN communication is shown on the right side.

The following subsections detail the two main partitions that compose DSSRCA: Reliable and Best-Effort protocols. Additionally, details regarding the implementation and some experimental results are also presented.

### 3.1. UAV-to-Sink Communication

DSSRCA provides a partition for performing a reliable communication between the UAV and the Sink. This layer makes use of 6LoWPAN [28] in conjunction with our customized transport layer, which aims at improving the reliability in terms of message transmissions.

The proposed protocol also allows to distinguish messages among three different classes of priorities: (i) low; (ii) normal; and (iii) high. The messages are stored in a priority queue implemented using binary heaps. For each priority class there is a maximum number of re-transmission attempts associated. High priority messages have an unlimited number of re-transmission attempts. Messages with medium priority have *n* attempts (typically 5), and messages with low priority have no re-transmissions.

To improve reliability, the proposed approach provides basic mechanisms for message re-transmission, duplicated packet detection, and error-checked delivery of a stream of octets between applications running on both the UAV and the Sink. It follows the philosophy of connection-oriented protocols, meaning that applications must first establish a connection with each other before they send data in full-duplex mode.

In order to guarantee proper data delivery, the protocol uses a technique known as positive acknowledgment, in which the sender transmits the message with its own sequence number, and waits for the receiver to acknowledge the message reception by sending an ACK message with the sequence number received. At the moment the sender transmits the data, a timeout mechanism is initiated to wait for the acknowledgment. If the timer expires before the message has been acknowledged, the sender re-transmits the message.

A very important point to ensure the data transmission efficiency, is the tuning of the re-transmission timer (RTO). If the value is too high, the protocol will be slow in reacting to a segment loss, thus reducing the throughput. On the other hand, if the value is too small, it will result in unnecessary re-transmissions that lead to exaggerated consumption of network resources. The goal is to find an RTO value that balances throughput degradation between both cases.

The proposed solution uses a dynamic algorithm to continuously adjust the timeout value based on monitoring the packet transmission delay. The round trip time variance estimation algorithm introduced by [29] was chosen and frequently applied in the TCP congestion control. The algorithm maintains a *SmoothedRTT* variable to represent the best estimated timeout for the packet round-trip time. When a message is sent, a timer is triggered to measure how long it takes to get the confirmation and stores the value in the *MeasuredRTT* variable. Then, the algorithm updates *SmoothedRTT* as shown in Equation (1).

(1) SmoothedRTT=α.SmoothedRTT+(1-α).MeasuredRTT

where *α* is a smoothing factor with a recommended value of 0.9: in each measure, *SmoothedRTT* is updated using 10% from the new estimate and 90% from the previous one.

In order to respond to large fluctuations in the round-trip times, RFC793 recommends using the re-transmission timeout value (RTO), as defined in Equation (2):

(2) RTO=min[UBOUND,max[LBOUND,β.SmoothedRTT]]

where *β* is a delay variance factor with a value of 1.3. The constant UBOUND is an upper bound on the timeout with a minimum value of 100 ms. The LBOUND is a lower bound on the timeout with a value of 30 ms. The values of *β*, LBOUND and UBOUND were obtained by conducting empirical tests on the UAV-to-BS communication. This mechanism can lead to a poor performance since the sender does not transmit any message until the acknowledgment of the last message is received. To solve this problem, a new solution based in the TCP sliding window concept [30] is proposed in this work. Sliding window is the number of messages the sender transmits before waiting for an acknowledgment signal from the receiver. For every acknowledgment received, the sender moves the window one position to the right and sends the next message. If the receiver receives a message outside the range of the window, the message is discarded. However, if the message is in the range, but out of order, it is stored. For each message, the sender starts a timeout as previously described.

When transmission losses occur, the proposed algorithm does not reduce the window size as is done by the traditional TCP congestion control algorithms. This is justified since the message loss is related to transmission errors and not to network congestion. Reducing the window size decreases the data delivery rate, and therefore the window size should be maintained or even increased. This technique is discussed in different studies, such as [31], in an effort to adapt TCP to wireless networks or other high error rate mediums.

The multiple segment losses in the window can cause a degradation effect in the data rate. Traditional TCP implementations use cumulative acknowledgment scheme, in which the receiver sends an acknowledgment meaning that the receiver has received all data preceding the acknowledged sequence number. This mechanism forces the transmitter to wait for the timeout to find out that the message has been lost, which causes a flow decrease. The proposed TCP SACK (Selective Acknowledgment) [32] increases TCP performance in networks with high loss rate. The SACK aims to recover multiple lost segments within one RTT. To achieve this, the receiver provides enough information about the segments lost in its sliding window. With this information, the sender knows exactly which segments were lost and re-transmits them before the timeout expires.

For implementing the sliding window algorithm with SACK, the sender maintains a set of sequence numbers corresponding to frames it is permitted to send, which are represented by two variables: S${}_{L}$ (sender lower limit), *i.e.*, the number of the oldest message sent but not acknowledged yet; and S${}_{U}$ (sender upper limit), *i.e.*, the number of the next message to send. Similarly, the receiver also maintains a receiving window corresponding to the set of messages it is permitted to accept, which are also represented by two variables: R${}_{L}$ (receiver lower limit), *i.e*., the lowest numbered message that the receiver is willing to accept; and R${}_{U}$ (receiver upper limit), *i.e.*, one more than the highest numbered message the receiver is willing to accept. The implemented sliding window algorithm with SACK is presented in Algorithm 1.

**Algorithm 1** Sliding Window Algorithm with SACK1:Transmit all messages in the sender’s window (S${}_{L}$ to S${}_{U}$-1);2:**if**
*Message arrives in the window and in correct sequence* ($R_{L}$) **then**3: Receiver acknowledges the message4: Receiver copy R${}_{L}$ to the application and advances its window5:**else if**
*Message arrives in the window but is out of sequence (sequence* > $R_{L}$
*and* <= $R_{U}$) **then**6: Receiver acknowledges the message for the highest message correctly received7: Attach a SACK list with the message(s) number(s) missing8:**end if**9:
10:**if**
*Sender receives an ACK for a message within its window*
**then**11: Mark this message as correctly sent and received12: If message number is S${}_{L}$ then increment S${}_{L}$ and S${}_{U}$ and transmit S${}_{U}$-113:**else if**
*Sender receives an ACK with a SACK list*
**then**14: Re-transmit the correspondent messages contained in the SACK list and restart the timeout15:**end if**16:
17:**if**
*Timeout Occur*
**then**18: Re-transmit the corresponding message19:**end if**

### 3.2. UAV-to-Sensor Communication

The second partition of DSSRCA aims at providing an efficient and small-footprint protocol to support the communication between the UAV and the WSN. To reduce the protocol footprint it is necessary to bypass the 6LoWPAN stack, which can be done by using Linux *raw sockets*.

**Figure 2:**
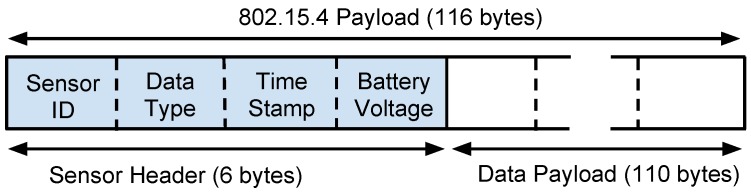
Message structure for Unmanned Aerial Vehicle (UAV)-to-WSN communication.

Such protocol is based on the frame format shown in Figure 2. *Sensor ID* is a number that identifies the message sender. *Data Type* specifies what type of phenomenon the sensor is observing. *Time Stamp* is used to identify the sensors local time of observation. Finally, the payload is the sensed data. The sensor header requires 6 bytes of the 802.15.4 payload size using short address mode, leaving 110 bytes for the data payload.

To collect data from the WSN, it is suggested that the network is organized in clusters [33], *i.e.*, there are cluster head (CH) nodes which collect and aggregate data from other sensor nodes in range. At the proper moment, the UAV triggers the CH with a *request message* for transmitting its data. In fact, this moment depends on the application under consideration.

Whenever a CH receives the *request message* and has data to transmit, it does so using unslotted CSMA-CA in the frame format previously described. When the UAV receives a message, an acknowledgment frame (ack), at the MAC level, is transmitted to confirm receipt. The CH continues to transmit until it has no more data or up to the moment it stops receiving the ack. Three attempts of re-transmissions are performed until the CH gives up transmitting. If this happens, the CH must wait for a new *request message* to re-start the transmission.

An important detail about this protocol regards the need for a modification in the UAV transmission power if compared to UAV-to-Sink communication. At the maximum UAV transmission power of +19 dBm, the CH would receive the *request message* even at a distance of hundreds of meters away from the UAV. In this case, the sensor would try to perform transmissions but would fail to reach the UAV due to the long distance. To tackle this issue, a mechanism named *Power Control* in the PHY level was implemented to switch the transmission power of the UAV radio to a lower level (–2 dBm) when communicating with the WSN.

### 3.3. Hardware Prototype

**Figure 3:**
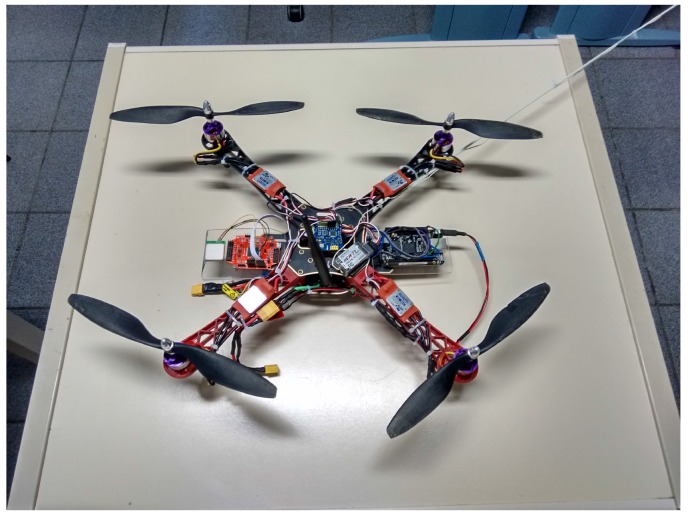
UAV equipped with the communication prototype.

In order to experiment with DSSRCA in real scenarios, a hardware prototype was developed and embedded in our UAV, as depicted in Figure 3. The core of the proposed solution is the MRF24J40MC transceiver (center-left part of the UAV), which implements an extended range version (and the standard version) of the IEEE 802.15.4 protocol. It was selected because the great majority of the WSNs are implemented using the IEEE 802.15.4 standard. It is a protocol suitable for connecting different devices at a short/mid-range, focusing on low-cost, low-speed (250 kbps), and low-power applications. The adopted radio transceiver contains a Power Amplifier (PA) and a Low Noise Amplifier (LNA) that allows communication ranges up to 1200 m. The adopted external antenna (at the center of the UAV) is a full wave +5 dBi antenna manufactured by Aristotle Enterprises. The antenna perpendicular, to the ground, is used for the 72 MHz RC.

The transceiver is connected to the embedded computing platform through a 4-wire SPI interface. As a computing platform, a low-cost credit-card-sized Linux computer equipped with a 1 GHz super-scalar ARM Cortex-A8 processor was selected (Beaglebone black [34]). The Ångstrom Linux distribution [35] is used as the OS. This embedded platform, located in the center-right of the UAV, also interfaces with a GPS and additional sensors/actuators.

### 3.4. Experimental Evaluation

This section aims to present practical experiments that make it possible to validate the proposed architecture, including its main ideas and the developed hardware prototype. Different experimental setups were created, in order to separately evaluate the two partitions that compose DSSRCA: Best-effort and reliable communication protocols.

The experiments were performed using two different types of mobile data collectors (MDCs): (i) a remotely piloted UAV and (ii) a bicycle. The main reason of using a bicycle equipped with our communication prototype is safety, as long-range experiments-related to the reliable protocol-were performed on a long and busy seaside walkway. On the other hand, the tests of the best-effort communication were performed in an open field using both the UAV and the bicycle.

The physical layer settings and other hardware characteristics of the experimental setups are presented in Table 1.

**Table 1:** Physical layer settings and other aspects of the experiments.

Parameters	Sink Node/UAV	Sensor
Radio Mode	Microchip MRF24J40MC	MICAz MPR2400CA
Antenna	Full-wave dipole	Half-wave dipole
Tx Power	+19 dBm (UAV-BS) –2 dBm (UAV-WSN)	–1 dBm
Automatic HW Re-transmission	3	3
Channel	11	11

#### 3.4.1. Best-Effort Protocol Experimentation

These experiment were performed using one wireless sensor (MICAz) deployed 70 cm above the ground level and a MDC. The MICAz sensor was configured to transmit 20 bytes of payload per message, with an unlimited number of messages in the buffer, whenever it was in the communication range of the MDC. Both, a remotely piloted UAV and a bicycle were used as MDC. Let us start by addressing the experiments using the UAV. The UAV was supposed to perform a rectilinear flight, approaching the sensor and departing from it at constant speed and altitude (see [36]).

**Figure 4:**
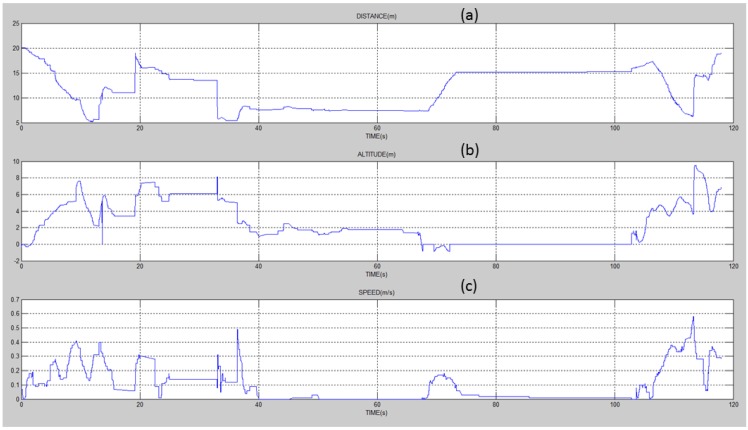
UAV experiment flight log with respect to (**a**) distance; (**b**) altitude; and (**c**) speed.

Figure 4 shows the flight data collected in one of the experiments, with distance at the top, altitude in the middle, and speed at the bottom. While the distance (to the sensor) is calculated by the UAV, altitude and speed are obtained from the GPS. As one can see, the altitude and the speed presented significant variations (while the original intention was to keep it constant). This implied changing the distance of the UAV to the sensor in a non-linear manner, making it impossible to obtain a proper estimate of the message reception rate with respect to the distance.

**Figure 5:**
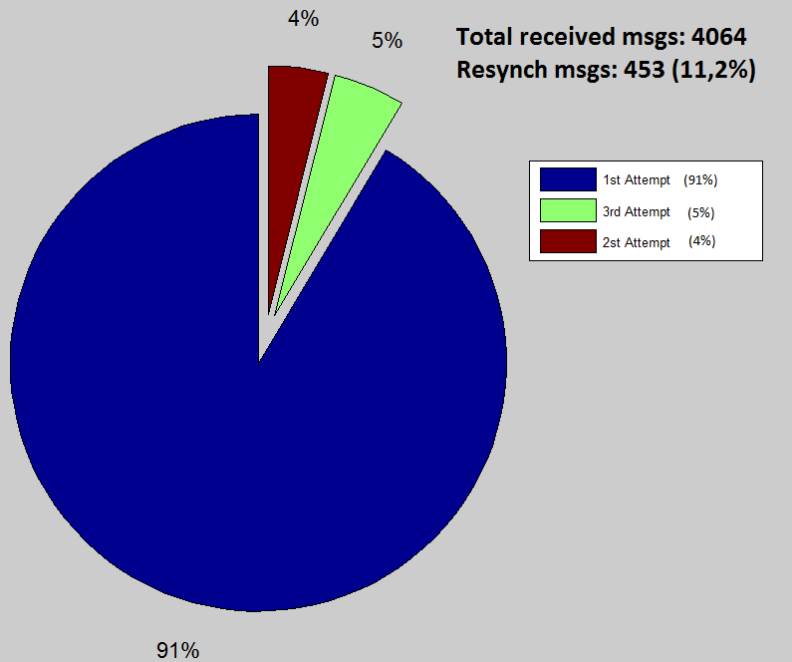
Messages received by the UAV along the experiment.

An interesting phenomenon to be observed is the action of the re-transmissions protocol at the sensor side. As stated before, it makes three re-transmissions attempts before stopping sending messages to the UAV. At this point, the sensor waits for a new *request message*, so that it can start transmitting again. Figure 5, presents the total number of messages received by the UAV during the 110 s of the experiment. The pie graph is used to divide the messages according to the number of re-transmissions necessary to deliver these messages. The results show that 91% are delivered in the first transmission attempt, which represents a very good transmission quality for the experimented distance. It is important to notice that 453 re-synchronization messages (*request message*) were transmitted by the UAV along the experiment. We consider this number to be higher than desired, so improving it will be the focus of further investigation.

These experiments allowed us to conclude that it is very difficult for an amateur pilot to perform a smooth flight trajectory. Only very experienced pilots or autonomous aircrafts would be able to achieve this goal, which would allow testing of the proposed communication prototype in a more suitable manner. Therefore, it was decided that further experiments use the bicycle.

In respect to the experiments with the bicycle, as previously mentioned, they make it easier for one to control both the bicycle path and its speed. The bicycle traveled on a rectilinear path so that it first approached the MICAz and then distanced itself away. The bicycle started at 125 m away from the sensor and stopped 125 m past it. The target speed was 7 m/s (see [37]).

**Figure 6:**
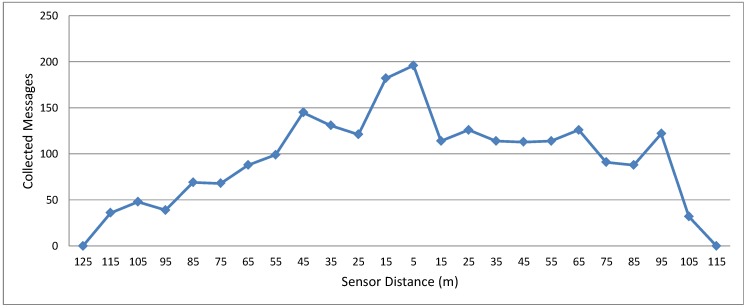
Histogram of messages collected by the bicycle.

Figure 6 shows the number of messages collected by the bicycle with respect to the sensor distance. This analysis is interesting in this experiment because the bicycle moves at a constant speed, without stopping. As expected, the distance directly affects the link quality. When the distance between bicycle and sensor increases, the number of messages collected by the bicycle decreases. Therefore, this experiment helps evaluate the distance that is most suitable to start collecting data.

**Figure 7:**
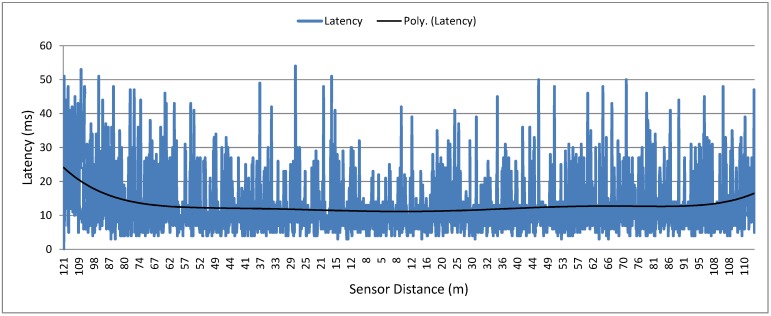
Sensor messages latency in the bicycle experiment.

The graph in Figure 7 shows the latency of the messages transmitted by the sensor. The measurement consists in the time difference between the moment the packet is sent to the transceiver upon receiving the ACK at MAC level. The dark line in the graph denotes the polynomial trend. It is possible to observe that the average message latency is about 15 ms from 5 to 80 m. Moreover, the latency increases at distances greater than 80 m since the link quality decreases and more re-transmissions are necessary. This experiment is important in order to highlight the boundaries of a good data collection mechanism. With this information, one can tune the protocol to optimize data gathering performance.

#### 3.4.2. Reliable Protocol Experimentation

As mentioned, these experiments were performed on a long seaside walkway. Besides natural obstacles such as poles and trees, it is in an urban area and the environment poses possible interference from other wireless technologies (e.g., WiFi)-which is good for testing the proposed re-transmission mechanism.

In this experiment, the MDC took about 9 min, at speeds between 4 and 8 m/s, to move 1400 m away from the sink and then return to it. It was configured to transmit one message per second. The message was composed of telemetry information with a payload of 32 bytes: 128 bits for the geographical coordinates and 128 bits for speeds north and west. The sink not only records the received telemetry data, but also transmits a ping message to the MDC to monitor the link.

**Figure 8:**
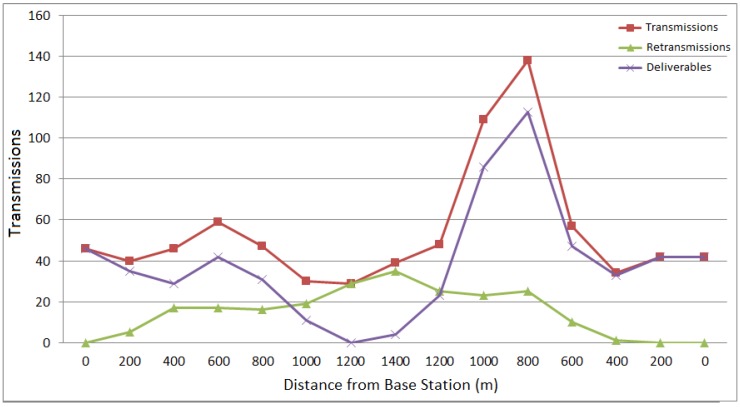
data collected from the reliable protocol evaluation.

During the experiment, 489 messages were transmitted by the MDC and received by the Sink. Figure 8 plots the three variables that are directly associated with the performance of the proposed re-transmission algorithm. The *Transmission* variable denotes the total number of messages that were transmitted, including those delivered and the re-transmissions attempts. The *Deliverables* variable is the number of messages successfully delivered and *Re-transmissions* is the number of failed re-transmissions that did not receive the ACK. The graph shows the average of these three variables at every 200 m.

It can be observed in the graph that in the first 900 m the number of delivered messages is considerably satisfactory. Then it started dropping until it reach its lowest point, contrasted with the increase in the re-transmissions (which reaches its highest level). When the MDC starts moving back there is a peak in the total number of delivered messages. This large number of messages includes those previously generated (and buffered), but that could not have been delivered due to the long distance from the sink.

It is important to note that the re-transmissions mentioned above are in the application layer, not in the link layer. The link layer re-transmissions can be performed automatically by the MRF24J40MC hardware using an *ack* at the MAC level. Such feature remained active during all of the experiments.

In order to measure the data rate, in another experiment we placed the MDC close to the sink and performed a 10 MB mission file download. This experiment was repeated several times using different block sizes. Whenever the 6LowPan receives a message (block) from the application to transmit (in this case at the Sink node), the message is typically divided into multiple fragments in order to fit the 127 bytes of the 802.15.4 maximum transmission unit (MTU). The MTU has 75 bytes available for payload in the first fragment and about 85 bytes for the remainder ones. A MAC-ACK is sent for any fragment correctly received in the destination node. If any single fragment is lost, it will not be re-transmitted, and all others already received will be discarded.

**Figure 9:**
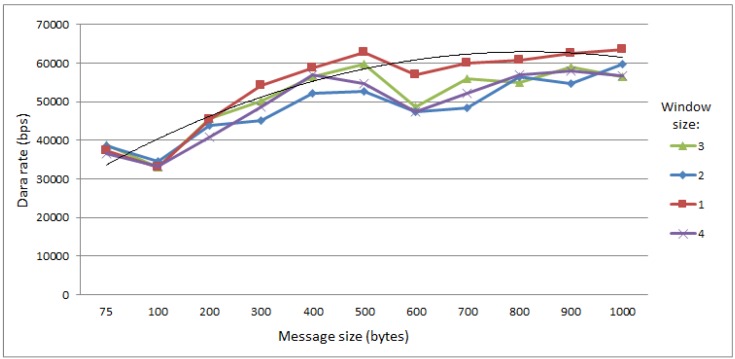
Data flow between mobile data collectors (MDC) and Sink.

It can be observed in Figure 9 that the data rate initially increases, since using bigger message blocks imply less confirmation messages at the application level. It is possible to notice a slight drop in the data rate from 500 to 600 bytes before it saturates. This comes from increasing the number of required fragments from 6 directly to 8, and increasing the number of fragments enhances exponentialy the probability of transmission failures. The tendency for saturation in the long run with transmission blocks bigger than 500 bytes can be observed in the tendency curve (the thin line that is not in the legend). In addition, it can also be observed in the figure that the use of windows size higher than one has a negative effect on the data rate. This occurs because when the UAV tries to transmit the application-level acknowledgment, the Sink tries to transmit the next message that is allocated in the transmission window. Multiple occurrences of such events increases the collision probability, which occasionally results in transmission losses.

**Figure 10:**
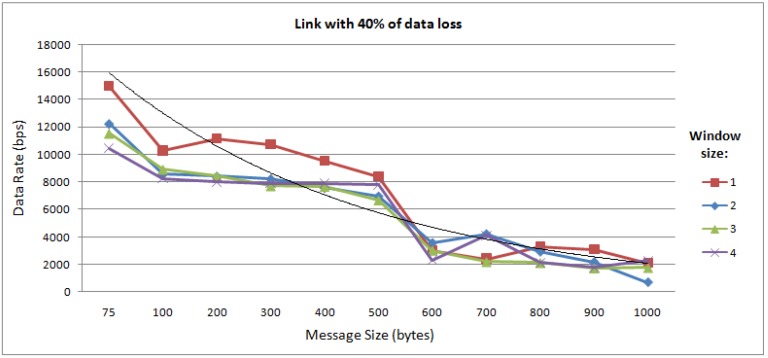
Data rate in the communication between the Sink and MDC, in a link with 40% loss probability.

The experiment was repeated using a link with a 40% probability of loss. It is apparent in Figure 10 that the data rate has behavior opposite to that of the previous experiment. The more the message size increases, the smaller the data rate becomes. This happens because bigger message blocks increase the probability of losing a single frame, which results in discarding all other frames already received by the 6LoWPAN. The same drop from 500 bytes to 600 bytes observed in Figure 9 is again observed here.

It should also be observed that if there is no loss in the network, it is desirable for the application to send bigger blocks because fewer acknowledgment messages at the application level are required. However, when there are losses in the network, it is more advantageous for the application to send messages of a smaller size, because the loss of a single frame does not discard the frames already received.

**Figure 11:**
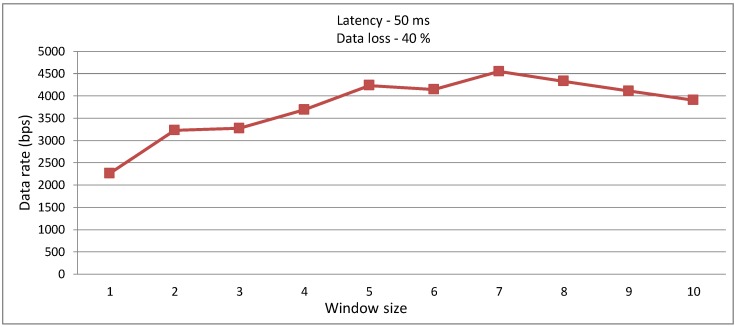
Data rate in the communication between the Sink and MDC, in a link with 40% loss probability and 50 ms network latency.

In the next experiment, the message size was fixed at 75 bytes and the size of sliding window was changed with a link of 20% loss probability, in a network with 50 ms of latency. The results obtained are shown in Figure 11. It can be observed that increasing the window size up to the limit of seven, the data rate increases. Thus, it allows us to conclude that it is always advantageous to use a window size larger than one when there is latency in the network; otherwise, the number of collisions causes the data rate to decrease.

## 4. Extended Simulation Analysis

The simulation analysis presented in this section intends to validate one of DSSRCA’s features, which is the fact that it uses only one radio for communication. Therefore, the experiment simulates a UAV acting as mobile data collector (MDC), which collects data from a WSN and then forwards it to a Sink node.

**Figure 12:**
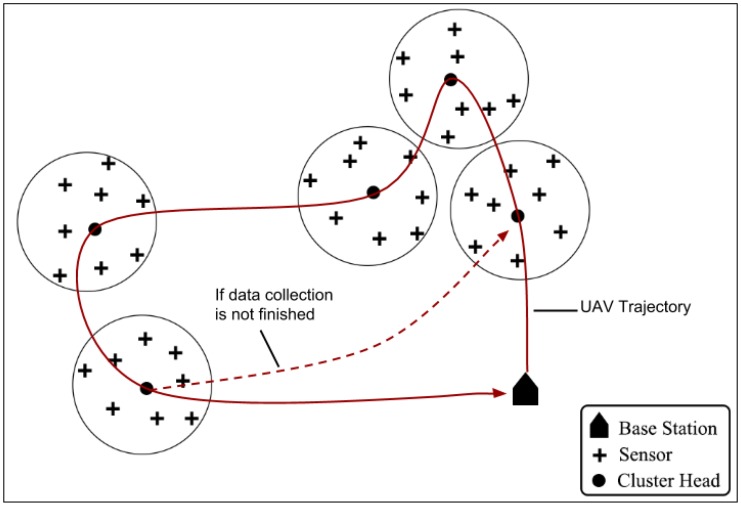
Simulation scenario: UAV flies over 45 nodes organized into five clusters.

It considers a practical example of an environmental monitoring system where sensors, organized into non-overlapping clusters, are deployed in a remote area. The sensor nodes sense their environment and save data in their buffers. Each sensor transmits data to its corresponding cluster head (CH), which in turn saves it and eventually send it to the UAV whenever it is in communication range. The cluster heads are responsible for coordinating the operation of the cluster members and also for communication with the UAV. A simulated environment of this scenario was developed in Omnet++ 4.41 with Inetmanet-2.0. The simulated environment was 1000 m × 1000 m. The altitude of UAV was kept constant at 15 m above the ground. We used 45 sensor nodes, organized into five clusters on the basis of distance from each others. Such simulation scenario is depicted in Figure 12.

To perform this experiment the following reasonable assumptions were made:

– A Sink node knows the location and propagation model (communication range) of all the ground sensors.
– The UAV is equipped with GPS, so that it can calculate its current position.

The transmission range of each node, including the CH is approximately a circle with 90 m radius. The radio model used for all nodes including UAV is 802.15.4. The UAV can make use of the extended transmission power (+19 dBm) to send data (telemetry or collected) back to the Sink, while traversing the network area. Before starting data collection, the Sink generates a flight plan for UAV, as it has all the necessary information regarding locations and radio ranges of sensors. The length of each data collection lap is approx. 1650 m. It should be noted that when the UAV starts flying, it already knows what trajectory to follow. The UAV can always estimate the start and end points of each CH zone. It is also assumed that the UAV trajectory passes over the CH node according to the flight plan generated by the Sink. The mechanism of this initial flight plan generation and its optimization is not considered here and is out of the scope of this work. We also do not consider how the sensors within each cluster behaves and communicate with CH. We assume that the sensor nodes remain silent (go into sleep mode) whenever the UAV enters the communication range of a CH. These sensor nodes remain in sleep mode until the UAV goes out of the coverage zone of that particular CH. This helps to avoid message collisions and disturbance in CH-to-UAV communication. This sleep/wake-up coordination is simply achieved by cooperation between UAV and CH. Whenever a UAV is about to enter a CH coverage zone it announces its speed while contacting the CH. Knowing his coverage area, the CH can easily calculate the time, the UAV is about to spend in communication range and instruct the sensors to go to sleep mode for at least that duration.

After the generation of the flight plan, the UAV begins traversing the network area and starts collecting data. As the UAV knows the location of each CH, when it enters the communication range of CH1, it asks CH1 to start sending its data. In case, the UAV does not know the location of each CH, a three-way handshake could be used for identifying the underlying CH.

In this experiment, the UAV was tested at different speeds, starting from the Sink on a pre-defined path, covering a total distance of 1650 m and returning back to the Sink again. In first case, the UAV is configured to only collect data from ground sensors and does not communicate with the Sink at all. In later cases, the UAV is configured to send one message of 70 bytes periodically after 200 ms, 500 ms, 1 s, 2 s, 3 s, 4 s, and 5 s to the Sink using its extended transmission power. The message sent by the UAV could be a telemetry packet or data collected from ground sensors.

**Figure 13:**
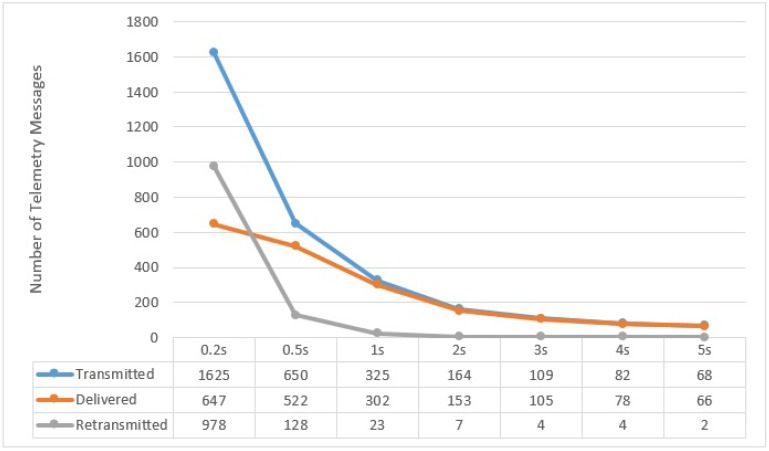
UAV to BS Communication at 5 m/s with respect to different Flush Back Periods.

**Figure 14:**
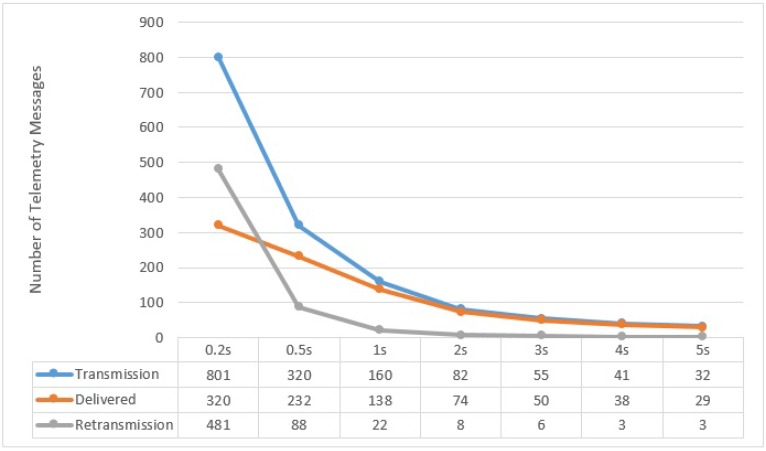
UAV to BS Communication at 10 m/s with respect to different Flush Back Periods.

**Figure 15:**
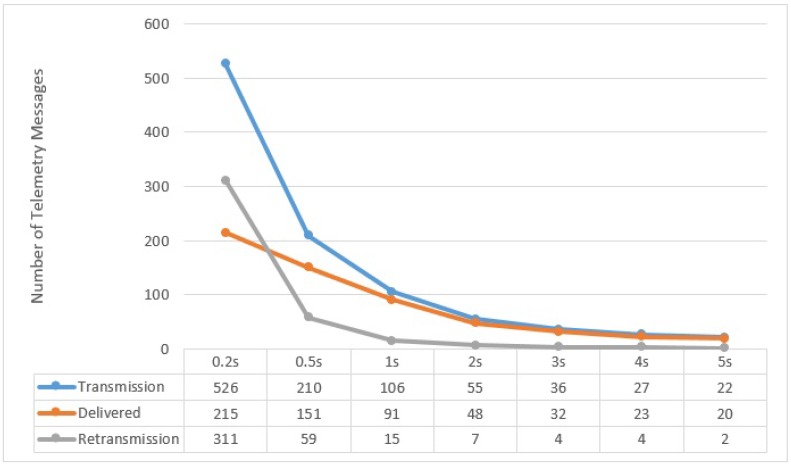
UAV to BS Communication at 15 m/s with respect to different Flush Back Periods.

All along the path of one complete lap of 1650 m at speeds of 5, 10, and 15 m/s, different numbers of messages were transmitted by UAV to the Sink, depending on the size of period the UAV used (e.g., 200 ms, 500 ms, 1 s, 2 s, 3 s, 4 s and 5 s) which we call the **Flush Back Period**. In this case, we present in Figure 13, Figure 14 and Figure 15, the averages of the *transmitted*, *delivered*, and *re-transmitted* variables. The *transmitted* denotes the total number of messages that were transmitted, including delivered and re-transmissions attempts. The *delivered* variable is the number of messages successfully delivered and *re-transmitted* is the number of failed re-transmissions that did not receive the ACK.

**Figure 16:**
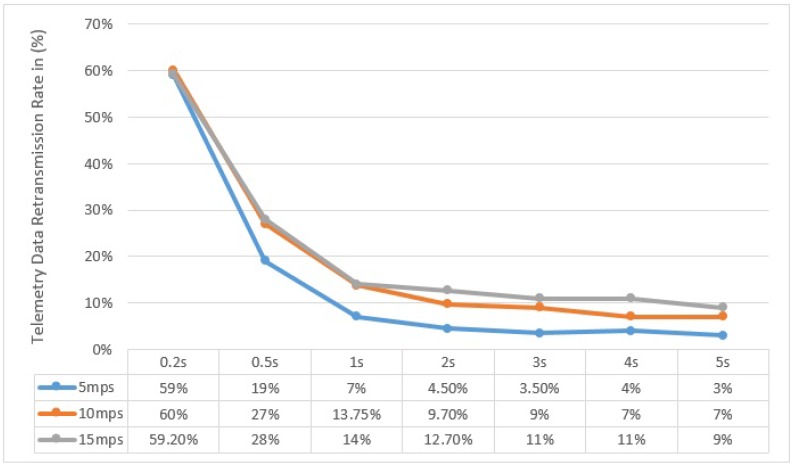
Re-transmission Rate of UAV to BS Communication for different UAV speed and Flush Back Period.

Similarly, the re-transmission rate of telemetry data (sent by UAV to BS), with respect to the speed of UAV and Flush Back Period, is shown in Figure 16.

It can be noted here that the successful delivery rate (from UAV to BS) increases with decreasing the UAV Speed and increasing Flush Back Period. The majority of the re-transmission attempts are in areas where the UAV is furthest from the BS. This behavior is also confirmed by our practical experiment shown in Figure 6. It must be noted that we do not consider how cluster members communicate with CH, although the communication between cluster members and CH is very important and it can affect the communication between UAV and CH. As described before, we use a simple and intelligent way for the CH, knowing the UAV speed and hence its approximated contact period, to instruct all non CH nodes to remain in sleep mode until the UAV is gone.

To measure the goodput, we repeated the simulation experiment several times with different UAV speeds and with a different Flush Back Period for one complete lap of 1650 m. It is assumed that each of the cluster Heads has infinite data to transmit to the UAV. In the first case, the UAV is configured to only collect data from the underground sensors and not to communicate with the Sink at all. In later cases, the UAV is configured to send one message of 70 bytes periodically after 200 ms, 500 ms, 1 s, 2 s, 3 s, 4 s, and 5 s to the Sink.

**Figure 17:**
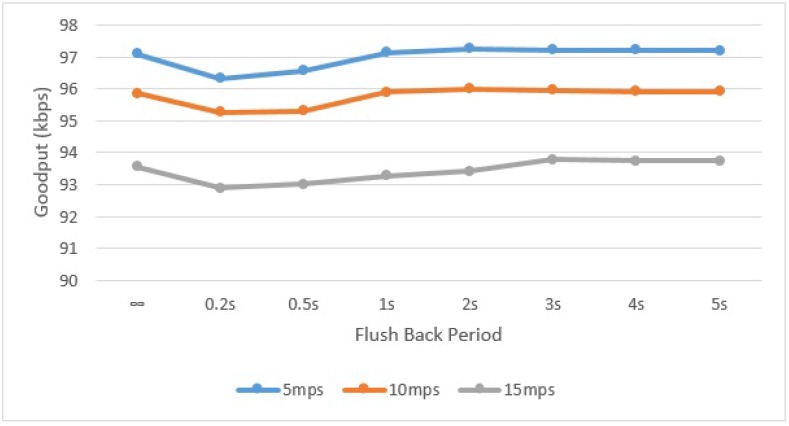
Network goodput for different UAV speeds and Flush Back Periods.

A very slight increase in the network goodput can be seen in Figure 17, when the UAV also sends data back to the Sink. This is due to the additional messages the UAV sends to Sink. In the case of short Flush Back Periods, the data rate is slightly decreased because the UAV spends more time sending messages to the Sink. It can be seen here that the data rate with and without sending telemetry data is roughly same. This shows the viability of using a single transmitter for a mobile data collector in a WSN. Furthermore it can also be noticed that the larger the distance or gap between clusters of sensors, the higher the goodput. The gap between sensor clusters, in which the UAV is not busy communicating with the underlying CH, can be used to flush collected data back to the Sink, and hence increase the goodput. In this way, an intelligent mechanism can also be implemented to vary the Flush Back Period so as to use the available bandwidth wisely.

It should be noted that the UAV and Sink talk on a communication channel other than the one used for communication between UAV and sensor network. This helps reduce the number of message collisions and disturbance in other part of the sensor network, as the UAV uses increased power for communicating with the sink.

In our simulation experiment, we assumed that the UAV can estimate its current location along its trajectory and can calculate when it will enter communications range of each CH while traversing the network area. This knowledge can be exploited by configuring the UAV to flush back its collected data when it is not in the range of any underlying CH. In this case, the UAV does not simply send telemetry data periodically, but uses full-fledged transmission to send back collected data to the Sink. The same simulation was repeated with this configuration for the UAV using Algorithm 2. In this case, the Network Goodput increases to 158.5 kbps when the UAV moves at 5 m/s, to 156.16 kbps when the UAV moves at 10 m/s and to 153.9 kbps when the UAV moves at 15 m/s.

**Algorithm 2** Full-Fledged Transmission from UAV to BS when free (not in the range of any cluster head)1:**procedure** Continuously Estimate UAV Current Location2: **while**
*UAVCurrentLocation* does not lies in a CH Range **do**3:  Send collected data back to BS4: **end while**5:**end procedure**

A marginal increase in the bit rate can be seen here. This is due to the fact that in this case the UAV is busy communicating throughout its trajectory. In this particular example, more than 75% of the collected data is sent back to the Sink before completing an entire round. As pointed out earlier, the larger the gap between sensor clusters, the more spare time the UAV has, and the more data it will send back to the Sink. In dense sensor networks or WSNs in which the sensors clusters are overlapped or nearly overlapped, we would not see this significant increase in data rate, because the UAV would find very little time to flush data back to the BS. Nevertheless, in this case, the UAV could still communicate with the BS using an appropriate flush back period.

## 5. Conclusions and Future Work

In this paper, we presented a dual stack single radio architecture that can be used in mobile sinks, specifically an Unmanned Aerial Vehicle (UAV), to address the problem of data collection in static, ground, wireless sensor nodes. We discussed how a low cost, dual stack protocol can be used by a short range UAV (or any other mobile device) to collect data from a WSN. Moreover, the viability of using a single radio transmitter for simultaneously collecting data from the WSN and communicating with the Sink node or Base Station was shown. Our architecture design allows the UAV to collect data from heterogeneous ground sensor devices and send telemetry or collected data back to the Sink using the same communication hardware and without compromising the data rate. This prototype design has clearly established the feasibility of using a single communication hardware for mobile nodes for data gathering in sensor networks.

This paper also detailed the proposed communication protocol, which is capable of dealing with two different requirements: a best effort communication between the UAV and the WSN and a more reliable communication to be used between the UAV and Sink. This paper presented the successful use of the proposed architecture within an environmental monitoring application scenario. It can also be observed that the proposed dual stack architecture is not only valid for the UAV, it is also generic enough to be used for ground mobile nodes as well.

In this work, we did not focus on how sensor nodes communicate with the CH, although in a real application this needs to be configured in a way that sensor-to-CH communication does not collide with CH-to-UAV communication. Different sleep/wake-up pattern can be used for such purposes. It is also possible to exploit cooperation (information sharing) between UAV and ground nodes to avoid messages collisions. Similarly, it is also possible for static nodes, particularly the CH, to learn and predict the mobility pattern of UAV to further enhance UAV detection. This will not only decrease message collisions, but also enhance network lifetime and allow more accurate sleep/wake-up patterns for sensor nodes. This along with effect of single radio transmitter on the lifetime of network needs to be further addressed.

Future work also includes removing the UAV remote pilot by designing and implementing a dynamic speed control for the UAV, in which speed is adaptively and autonomously adjusted according to the application needs. Similarly, a dynamic Flush Back Period is also under consideration so as to use available bandwidth wisely. Finally, additional practical experimentation alternating best-effort and reliable transmissions should be performed.
